# Diseases of the Prostate Gland and Their Treatment

**Published:** 1883-07

**Authors:** Ad. Lippe

**Affiliations:** Philadelphia


### THE DISEASES OF THE PROSTATE GLAND AND THEIR TREATMENT.

AD. LIPPE, M. D., PHILADELPHIA.

The most frequent diseased conditions of the prostate gland are acute inflammation, and, as a sequel, chronic inflammation, hypertrophy, obstructions, and tumors ; less frequent are tubercles, cancer, and stones of the prostate gland.

The acute inflammation is, with rare exceptions, a disease of early manhood and is caused by the suppression of gonorrhœa, by the abuse of Cubebes, Balsam of Copaiva, by venereal excesses, by the abuse of alcoholic liquors, by riding on too hard a saddle, or by blows on the perineal region.

The symptoms of this disease are a sensation of heat and pain in the perineum, with fullness and throbbings like those of the pulse; an incessantly renewed desire to pass small quantities of water ; and having emptied the bladder no relief is obtained from these useless efforts (vesical tenesmus). The urine passing that part of the canal surrounded by the prostate gland produces a very vivid sensation of burning, the seat of which is referred to by the patient as the neck of the bladder. The rectum seems to be filled by a large, heavy body which increases the desire for evacuation, forces the exercise of this function, and urges the patient to continue the efforts even when the evacuations have been complete. By introducing the left index finger into the anus great heat and painfulness to pressure is perceived, also a smooth, round, hot tumor, making a lump in the intestines of a very considerable size. When the swelling of the gland has caused an occlusion of the canal and thereby a complete retention of urine, catheterization becomes *apparently* necessary, but it is not always successful. The instrument passes with facility through the two anterior parts of the canal (the spongy and membranous portions), but the passage through the prostatic portion causes a very acute pain, sometimes even unbearable. If you pass your index finger into the anus while the instrument is in the bladder the prostate gland is found between the instrument and the finger, and its volume can so be judged.

The acute inflammation is generally of a very rapid course and usually runs through all its stages from six to eight days. The more favorable termination is resolution, but if not properly treated the disease passes over into the chronic state (infiltrations, tumors) or an abscess forms, which opens finally in the urethra, the bladder, or the rectum, and leaves prostatic fistulas. In very rare cases the inflammation of the prostate gland terminates in gangrene.

The formation of an abscess causes an increase of all the symptoms present; the frequent painful stitches which had been felt from the perineum to the regio pupis and from there occasionally shooting down the thighs give way to violent throbbing pains. The opening of the abscess is followed by a sudden relief of the pains and sufferings.

Chronic Inflammation, Hypertrophy, and Tumors of the Prostate Gland.

The prostate gland may be so affected in its totality or only one or the other of its lobes. The swellings, tumors, and obstructions of the prostate gland in whole or in part are the consequences of an acute inflammation of the gland. They develop themselves very slowly, sometimes in the progress of years. These tumors also result from an excess of nutrition (hypertrophy) or from deposits of foreign matter, tubercles, cysts, pus, fibrine, and fibrous bodies in the cellular tissues of the organ. The prostate gland of an adult in the normal state is of the form and the size of a French chestnut. Under the influence of different causes it can attain the size of a hen's or turkey's egg or even of the head of a man, as Bartholow mentions a case.

Every swelling and enlargement of the prostate gland disturbs and modifies the mechanical functions of the urethra, especially those of the pars prostatica, of the neck of the bladder, those of the ductus ejaculatorii; the size, the direction, the length, and the passage of these ducts and channels become changed. If the prostatic gland has increased in volume it necessarily ascends upward and backward in the cavity of the pelvis. This ascension of the swollen prostatic gland accounts for the high position of the neck of the bladder in old persons and for the increased length of the urethra, so that often the longest sound will scarcely reach the bladder. As the prostate gland tightly incloses the neck of the bladder (the pars prostatica urethræ) the bladder is compelled to ascend with the gland.

The more apparent is the mechanical disproportion from the abnormal development of the middle lobe as it appears most frequently during old age. This middle lobe seems either as a round, flat roll which is more or less thick, and standing out against the Trigon-Lieutaudii, or in the shape of a stalked swelling, movable like a flap, or else it forms, by pressing forward fold-like the muscles and mucous membranes by which it is covered, a swelling which rises from the two side lobes over the middle lobe and becomes an oblique and upright or even perpendicular ridge which closes, according to its height, either entirely or partially, the neck of the bladder from within and in part or fully prevents the passage of the urine. The ridge (Barriere urethro vesicale) is so important in the diseases of the bladder in old persons, that finally, in later days, Mercier has had the merit of having clearly demonstrated the anatomical relation of this very important anomaly. If one of the side-lobes becomes enlarged it causes a curve of the pars prostatica urethræ corresponding with the degree of the tumefaction; when both side-lobes are equally swollen, the urethra becomes compressed from both sides, so that the canal forms a compression only behind and before and somewhat gaping. The swollen lobe of the prostatic gland sometimes presses in the shape of a blunt conical peg in the cavity of the bladder and thereby closes from one side the neck of the bladder or gives it an oblique direction. At times one or the other of these peg-like swellings forms a sort of thick flap which is the tighter pressed against the neck of the bladder by the flow of the urine. The reverse may happen through a diverging direction of the swollen lobe; the neck of the bladder then suffers a funnel-like elongation, and the extension paralyzes the sphincter.

The most prominent symptoms are, discharge of prostatic fluid during a stool, diminution of the stream of urine, frequent necessity to empty the bladder, very great difficulty and often impossibility to do it; when the patient is necessitated to urinate it takes some time before he can commence; once flowing, the urine continues abundantly enough but in an unequal stream or in a dripping manner. In spite of the greatest efforts, the bladder cannot be completely emptied, and if the patient is sounded immediately after micturition a great deal of liquid is yet found in the bladder. The urine sometimes escapes drop by drop and involuntarily, often accompanied by obstinate constipation.

All these symptoms occurring in the same patient indicate with almost a certainty the presence of a prostatic tumor, but to be assured not to mistake it with other analogous diseases of the urinary organs, it is well to proceed to an examination by the double exploration of the finger and the sound.

In the treatment of any of the above described abnormal conditions of the prostatic gland, the true physician will never be guided by the name of the disease or by the pathological condition of the diseased organs in the choice of the remedy; it will not matter whether the inflammation is chronic or acute, the true physician, knowing that *the totality of symptoms alone constitutes the disease*, will select the remedy which is most similar in its effects to the symptoms of his patient, and while the various abnormal conditions of various organs and parts of the body will present characteristic changes and symptoms, these symptoms are in every case more or less modified by the conditions and peculiarities of the patient attacked by the abnormal condition of the organ; making, therefore, each case to be treated as a special case. No one familiar with Homœopathy can believe in specific medicines for specific diseases, or contend, for instance, that "Aconite" will cure all diseases, that a certain trituration of Crotalus will cure all cases of yellow fever, or a certain trituration of the Protiodide of mercury all cases of diphtheria; such a belief would be becoming the uninitiated; and experiment, the infallible judge in medical and other theories, would convince any person of moderate judgment and ability that such generalizations are detrimental to the practice of medicine. All and each abnormal condition of a diseased organ show among the symptoms produced some characteristics, just as every medicine will produce characteristic symptoms which *we* know are produced on all provers. While many specialties are often only the effects produced on one individual, this specialty may again in turn correspond with one of a diseased organism and be the means of indicating the curative medicine.

When we ascertain the characteristic symptoms of a so-called disease or form of disease, we necessarily will find among the known medicines, *i. e.*, known to us by having been proved on the healthy organism, some few medicines most similar in their action on the organism to the characteristic symptoms of the disease. These medicines will, therefore, often cure that disease; but, if other symptoms besides the ordinary characteristic symptoms have been developed by the peculiarities of the patient, then we have to employ such medicines as correspond with the disease of the patient and *his* peculiar symptoms, and therefore it remains true that in all diseases all medicines may be employed.

The diseases above referred to, of which I have tried to give the characteristic symptoms, may very often be cured by the following medicines:

Pulsatilla, Thuja, Digitalis, Cyclamen, Selenium, Causticum, Lycopodium, Secale cornutum, Copaiva, Zinc, Agnus cast., Alumina, Hepar, Apis, Sulph. acid.

*Pulsatilla.*—In Hahnemann's *Materia Medica Pura*, Vol. II, we find symptoms 466, 488, 489, 490, 493, 495, 497, 499, 500, 501, 503, 519, 524, constituting a strong picture of disease of the prostate gland. The pain in the region of the bladder, the frequent desire to urinate, but especially the continued dull stitch in the neck of the bladder, with a pressure of urine while lying upon his back, having to urinate very soon, and not while lying on the side; and when symptom 466, which Hahnemann records in parenthesis (the stool is of a small shape and as if pressed flat), and which was the first Pulsatilla symptom according to which this medicine was administered in the diseases of the prostate, where no remedy was known to the medical world having a specific effect in this disease and until then considered beyond the reach of all medicines;—all these symptoms will in many cases point to Pulsatilla; and another still more important symptom taken from the curative effects of the remedy, after micturition spasmodic pains in the neck of the bladder, extending to the pelvis and thighs, will often lead to the administration of this very important medicine in such disorders.

*Thuja.*—We find among Hahnemann's symptoms the following:

131. Frequent urinating almost every hour, but without pain.

134. When the patient wishes to urinate he strains much, the desire is felt every minute, the urine passing only now and then, and at that time only he has a burning pain in the urethra.

135. The stream is interrupted five or six times before the bladder is entirely emptied.

147. Stitches in the urethra starting from behind when not urinating, but not during the discharge of urine.

148. A violent stitch from the rectum into the urethra.

156. Discharge of prostatic fluid (stringy) in the morning, after awakening.*

159. A cutting pain immediately before and during the discharge of urine, most violent, in the region of the bladder, back of the pelvic-bone while walking.

Dr. Carl Myerhoffer in his report of the Vienna Provers has the following symptoms:

Pressure on the neck of the bladder.

Painful stitches from the anus to the orifice of the urethra or in the reverse direction.

Discharge of glutinous mucus from the male urethra.

Discharge of liquor prostaticus.

Burning stitches in the penis as far as the testicles.

Dr. C. Wolf in his most excellent provings of Thuja gives:

527. Frequent pressing to urinate with small discharges.

529. Continued desire to urinate.

533. The urine comes frequently with a tension as from a cord over the bladder, which seems to arrest the passage of the urine.

541. Urine with interruptions, and the urine passing only at intervals.

560. Frequent stitches in the urethra with a mucous discharge in the morning.

518. Sensation in the rectum as though a bladder had formed.

From clinical observations I add that the most frequent desire to urinate comes on from five till nine P. M., and is much relieved by a recumbent position.

All these symptoms are frequent attendants of the various diseases of the prostatic gland. But while Pulsatilla will more frequently correspond to the purely inflammatory condition and to the recent cases, Thuja will very often remove the disease permanently, especially when the disorder originated in syphilis, sycosis, and again more especially when gonorrhœa was the origin of it. Even in cases where already suppuration of the indurated gland had been established, and after repeated abscesses had discharged, leaving the gland in a general suppurating process, Thuja will often much re-

* This and the following symptom have been left out of *The Symptomen Codex* by the compiler.

lieve the suffering and retard the progress of the disease. In cases where the syphilitic origin is not present, and in the induration or hypertrophy of the gland, often occurring in the advanced age of man, Thuja will but rarely find its field of action. In those cases, not the result of or connected with syphilis or gonorrhœa, we will then often find an invaluable remedy in

*Digitalis purpurea.*—In the third volume of Hahnemann's *Chronic Diseases* we read the following symptoms which give us a very accurate indication in some cases of hypertrophy and induration of the prostate gland:

415. Retention of urine.

416. *Pressure on the bladder* with a sensation of great fullness, which continues after passing urine.

419. Continued desire to urinate even after passing the urine.

420. Violent fruitless effort to urinate.

421. Continued desire to urinate, with but a scanty discharge each time.

422. Uninterrupted desire to urinate, with the discharge of but a few drops at a time.

425. Continued desire to urinate and when rising giddiness from it.

434. Incontinence of the urine.

The symptoms 416, 419, 420, 421, 422, and 425 are of frequent occurrence in the hypertrophy of the prostatic gland and will yield very readily to the smallest dose of Digitalis. From my own clinical observations I can add a few more symptoms of Digitalis.

Before urinating and while the ineffectual pressure is causing violent agony, there is a throbbing pain as from a pulse in the region of the neck of the bladder. Before and after passing urine, violent stitches from the neck of the bladder to the end of the urethra. The desire to urinate, the pressure and suffering, increase after a scanty discharge of urine, compelling the patient to walk about in great distress until after some time another scanty discharge takes place.

The Digitalis urine is generally pale but slightly cloudy, looking smoky. Motion increases the desire to urinate, but when it once exists the patient cannot sit still nor lie down, but walks about in agony, gritting his teeth and despairing. The sleep is often interrupted by these periodically returning attacks, the desire to evacuate the bowels often accompanying the paroxysms; very small soft stools are frequently passed without relief, the pressure in the rectum continuing.

*Cyclamen.*—This near relative of Pulsatilla has very important symptoms which may at times indicate it in these diseases.

In Hahnemann's *Materia Medica Pura*, Vol. II, we find symptoms

86. In and near the anus and in the perineum, drawing pressing pain, as from sub-cutaneous ulceration of a small spot, while walking and sitting.

91. Frequent desire to urinate, with but a scanty emission of urine.

92. While urinating prickling pain at the end of the urethra.

*Selenium.*—The following symptoms found among the provings of this remedy may at times indicate it in this disease.

While sitting, and also while walking, a drop of viscid, transparent fluid presses out of the urethra, occasioning a peculiarly disagreeable sensation; the same sensation is experienced shortly before and after stool.

In the diseases of aged persons it may be at times of much benefit.

*Causticum.*—We find in Hahnemann's *Chronic Diseases*, many symptoms analogous to these diseases; he gives

721. Strong pulsations in the perineum.

722. Pains in the bladder; the patient passes no urine, and if a few drops come there is violent pain in the urethra, with constipation and spasms in the rectum.

723. Ineffectual effort to urinate, and when a few drops are passed there is violent pain in the bladder; when he walks much to relieve himself he has spasms in the rectum.

724. Desire to urinate, without being able to pass anything; after waiting very long but a little is discharged, and the pressure soon comes on again, without pain.

*Lycopodium.*—Among many analogous symptoms we find in Hahnemann's *Chronic Diseases.*

315. While passing urine, pressing in the perineum, near the anus, which continues, and often returns while not urinating.

321. Stitches in the neck of the bladder and at the same time in the anus.

*Copaiva balsam.*—This remedy is reported to have cured induration of the prostate gland. The indications for its application we take from the provings.

Constant, inefficient desire to urinate.

The urine is emitted by drops.

*Apis mellifica.*—The great analogy between the ovaries and the prostate gland make it very probable that Apis will exert its beneficial influence as well over the prostate gland as it does over the ovaries. When we take in consideration the effect Apis produces according to provings as we find in *The Amerikanische Arzneiprufungen*, by Dr. C. Hering, and in these provings symptoms—

646. A very disagreeable sensation in the bladder with a bearing downward in the region of the sphincter and a so frequent desire to urinate that the patient had not only to pass urine very often during the day, but that it was necessary to rise during the night from ten to twelve times for that purpose; during micturition much burning and smarting;

647. An almost incessant desire to pass urine;

652. Frequent desire to urinate with a disagreeable sensation in the bladder, a pressing down in the region of the sphincter;

673. Pricking in the urethra;

643. Retention of urine so that the urine had to be taken away by the catheter—

we can expect with a great degree of certainty that Apis will cure some cases of prostatic diseases. Without further quoting the sources that would guide us in the administration of many more remedies, I shall conclude by stating the most prominent symptoms and the corresponding remedies:

Discharge of prostatic fluid during a stool, Agnus c., Alum., Anac., Calc. c., Carbo veg., Con., Elaps, Hepar, Ign., Natr. carb., Sep., Sil., Staph., Sulph., Zinc.

Fullness in the perineum, Alum., Berb., Bryonia, Cycl., Nux vom.

Heaviness, sensation of, in the perineum, Copaiva, Graph.

Pulsation in the perineum, Caust.

Continued desire to urinate, Amm. c., Amm. m., Anac., Apis, Asar., Aur., Bell., Canth., Colch., Cop., Digital., Guaj., Ignat., Iod., Merc., Milleff., Mur. acid, Phos., Puls., Sep., Scill., Sulph., Sulph. acid, Thuja.

Impossible to urinate, Digit., Sepia.

The desire to urinate continues after micturition, Bar. c., Bov., Calc., Caust., Carb. an., Croton tig., Digital., Guaj., Nat. carb., Ruta, Thuja, Zinc., Bry., Lach., Merc., Sabad., Staph., Viol. tr.

While urinating burning in the region of the neck of the bladder, Cham., Nux v., Petr., Sulph. acid.

The stream of the urine is small, Graph., Nit. acid., Ol. an., Sars., Spong., Staph., Sulph., Tax., Zinc.

Difficulty in voiding the urine, and he must press a long time before he can commence, Alum., Apis, Hep., Raph., Sec., Tax.

Escape of urine involuntarily, drop by drop, Arnica, Bell., Mur. acid., Digital., Petr., Puls., Staph.

